# Pallidum Functional Hypoconnectivity and Inhibitory Control as Partial Mediators of Environmental Influences on Tobacco and Marijuana Initiation

**DOI:** 10.31586/jcn.2025.1140

**Published:** 2025-01-24

**Authors:** Shervin Assari, Hossein Zare

**Affiliations:** 1 Department of Internal Medicine, Charles R. Drew University of Medicine and Science, Los Angeles, CA, United States; 2 Department of Family Medicine, Charles R. Drew University of Medicine and Science, Los Angeles, CA, United States; 3 Department of Urban Public Health, Charles R. Drew University of Medicine and Science, Los Angeles, CA, United States; 4 Marginalization-Related Diminished Returns (MDRs) Center, Los Angeles, CA, United States; 5 Department of Health Policy and Management, Johns Hopkins Bloomberg School of Public Health, Baltimore, MD, United States; 6 School of Business, University of Maryland Global Campus (UMGC), Adelphi, MD, United States

**Keywords:** Socioeconomic Status, PM2.5 Exposure, Fine Particulate Matter, Resting-State Functional Connectivity, Inhibitory Control, Tobacco and Marijuana Use Initiation, Adolescents

## Abstract

**Background:**

Low socioeconomic status (SES) has been linked to higher rates of tobacco and marijuana use initiation; however, the contributions of environmental and neurocognitive factors remain underexplored. This study investigates a potential pathway connecting low SES, fine particulate matter (PM2.5) exposure, brain functional connectivity, and inhibitory control to increased tobacco and marijuana use initiation among adolescents.

**Objectives:**

To examine the mediating roles of PM2.5 exposure, resting-state functional connectivity between the right pallidum and the ventral attention network (P-VAN rsFC), and inhibitory control in the relationship between low SES and tobacco and marijuana use initiation.

**Methods:**

Data were drawn from the Adolescent Brain Cognitive Development (ABCD) study to assess associations between baseline SES, baseline PM2.5 exposure (based on zip code), baseline P-VAN rsFC, baseline inhibitory control, and subsequent tobacco and marijuana use initiation. Mediation models were used to determine whether PM2.5 exposure and changes in P-VAN rsFC act as pathways linking low SES to diminished inhibitory control and subsequent substance use initiation.

**Results:**

Low SES was associated with higher PM2.5 exposure, which, in turn, was linked to alterations in P-VAN rsFC. These alterations were correlated with lower inhibitory control, which significantly predicted tobacco and marijuana use initiation over time. Inhibitory control partially mediated the relationship between low SES and substance use initiation, indicating a complex pathway influenced by environmental and neurocognitive factors.

**Conclusions:**

This study identifies a potential mechanism linking low SES to tobacco and marijuana use initiation through environmental and neurobiological pathways. Understanding how PM2.5 exposure and neurofunctional connectivity impact inhibitory control can provide valuable insights for developing targeted interventions to reduce substance use among adolescents in low SES environments.

## 1. Introduction

According to biopsychosocial models [1-3], the social patterning of tobacco and marijuana use initiation in youth is complex [4,5], involving multiple pathways that connect environmental, neurobiological, and behavioral factors [6-10]. Tobacco and marijuana use initiation during adolescence is influenced by a range of elements, with socioeconomic status (SES) playing a key role in its epidemiology. Lower SES is consistently associated with higher rates of tobacco and marijuana use initiation among adolescents. This pattern may be partially explained by environmental exposures, such as fine particulate matter (PM2.5) [11], which are more prevalent in lower SES communities [12-15]. Neurobiological mechanisms also contribute to these outcomes, including low inhibitory control and altered resting-state functional connectivity (rsFC) between regions of the brain involved in reward processing and attention regulation. Understanding how these factors interact is crucial for identifying pathways that lead to early tobacco and marijuana use in adolescents.

One mechanism linking low SES to increased tobacco and marijuana use initiation involves exposure to PM2.5, a pollutant found at higher levels in low SES neighborhoods. Recent research has highlighted several pathways through which PM2.5 exposure can influence behavior, particularly through neurodevelopmental effects. Children from low SES families are more likely to live in areas with high PM2.5 exposure due to proximity to traffic, factories, and other pollution sources. In contrast, adolescents from higher SES backgrounds are less exposed to environmental risks like PM2.5, which can have significant implications for cognitive development and behavior [1]. Environmental toxins in lower SES areas can disrupt brain development, potentially increasing the likelihood of risk-taking behaviors, including tobacco and marijuana use. Exploring the mechanisms through which SES influences these behaviors is essential for crafting effective public health interventions.

Inhibitory control is a key cognitive function that can be measured both in laboratory settings and through self-reports. It plays a central role in regulating impulsive behavior and delaying gratification, both of which are critical for preventing risky behaviors like tobacco and marijuana use. Adolescents with lower inhibitory control are more likely to initiate substance use, including tobacco. Neurobiologically, inhibitory control involves the connectivity and function of several brain regions, such as the prefrontal cortex and the right pallidum, which interact with attention networks to govern self-regulation. Studies have consistently shown that reduced inhibitory control is a strong predictor of substance initiation and escalation, making it a vital focus in understanding adolescent tobacco and marijuana use.

The pallidum, a key component of the basal ganglia, is a subcortical structure involved in motor control, cognitive processes, and emotional regulation [16,17]. It includes the globus pallidus externa (GPe) and globus pallidus interna (GPi), each playing distinct roles in neural circuitry related to movement and behavior. Beyond its traditional role in motor control, the pallidum is crucial in regulating reward processing, decision-making, and impulse control. Its connections to brain regions like the prefrontal cortex and networks such as the ventral attention network are integral to inhibitory control, enabling the suppression of impulsive actions. Disruptions in the connectivity of the pallidum, especially with cognitive control networks, have been linked to impulsivity and substance use vulnerability. In adolescence, a developmental period characterized by ongoing brain maturation, changes in rsFC of the pallidum can have significant impacts on inhibitory control, contributing to behaviors such as early tobacco and marijuana use. The ventral attention network (VAN) is a critical neural system responsible for attentional control and detecting relevant stimuli, particularly unexpected or salient information. This network, which includes the temporoparietal junction and ventral frontal cortex, facilitates shifts in attention and adaptive responses to external cues. In adolescents, changes in VAN functioning can influence behavior, including risk-taking and substance use. Neuroimaging studies indicate that disrupted or atypical rsFC within the VAN is associated with reduced inhibitory control, which may increase the likelihood of engaging in risky behaviors like tobacco and marijuana use. Understanding how the VAN regulates attention is essential for identifying neural mechanisms underlying impulsivity and susceptibility to substance use, particularly in environments characterized by elevated stressors or pollutants like PM2.5.

Factors such as SES and PM2.5 exposure can impact the neural circuitry that supports inhibitory control, heightening the risk of impulsive behavior [18-21]. Consequently, inhibitory control may serve as a crucial mediator in the relationship between SES and tobacco and marijuana use initiation, underscoring the significance of both environmental and neurocognitive factors in shaping adolescent behavior. High PM2.5 exposure has been linked to changes in the functional connectivity of key brain networks and regions, contributing to lower inhibitory control and increased tobacco and marijuana use risk. Therefore, PM2.5 exposure may act as a neurobiological mediator in the pathway from low SES to tobacco and marijuana use initiation by altering neural connectivity and diminishing inhibitory control.

Based on research, we propose two primary connections between PM2.5 exposure and tobacco and marijuana use risk. First, PM2.5 exposure is strongly linked to low SES, putting disadvantaged populations at higher risk for neurodevelopmental changes that influence behavior. Second, PM2.5 exposure has been directly associated with changes in rsFC in brain regions crucial for self-regulation. Given these associations, a potential pathway from low SES to early tobacco and marijuana use among adolescents involves a series of connected factors: low SES leads to higher PM2.5 exposure, which then alters rsFC in the right pallidum and VAN, reducing inhibitory control and increasing the likelihood of tobacco initiation.

This study aims to explore a serial mediational pathway connecting low SES to tobacco and marijuana use initiation. We focus on the potential mediating roles of PM2.5 exposure, rsFC between the right pallidum and VAN (P-VAN rsFC), and inhibitory control. Unpacking this pathway may offer valuable insights into how socioeconomic and environmental factors intersect with neural mechanisms to influence adolescent tobacco behavior. These findings could inform targeted interventions aimed at reducing tobacco and marijuana initiation, particularly in low SES communities where environmental exposure and neurocognitive vulnerabilities are more pronounced.

## 2. Methods

### 2.1. Study Design and Participants

This study utilized data from the Adolescent Brain Cognitive Development (ABCD) Study [22-30], a large, longitudinal cohort study that follows over 11,000 children across the United States from ages 9-10 into young adulthood. The ABCD Study aims to explore the various factors—environmental, social, and biological—that contribute to adolescent development, including substance use behaviors. For this analysis, we focused on the baseline data collected from participants aged 9-10 years old to examine early factors influencing substance use initiation. The sample included youth from diverse socioeconomic and demographic backgrounds, providing a comprehensive overview of the early-life risk factors related to adolescent substance use.

### 2.2. Procedure

Participants and their caregivers / parents provided written informed consent and assent before participation. Baseline assessments were conducted at ABCD study [22-30] sites nationwide, including comprehensive surveys, neuroimaging, and environmental exposure assessments. Data collection procedures adhered to the highest ethical standards, with approval from Institutional Review Boards (IRBs) at each participating institution. Neuroimaging data were preprocessed and analyzed using standard pipelines provided by the ABCD study, ensuring consistency and reliability across sites. All analyses were conducted in accordance with ABCD guidelines for data privacy and participant confidentiality.

By using a combination of self-reported data, environmental exposure metrics, and neuroimaging assessments, this study aimed to elucidate the complex pathways through which socioeconomic and environmental factors influence adolescent substance use initiation.

### 2.3. Measures

#### Socioeconomic Status (SES):

Parental education was used as a proxy for SES in this study, categorized based on the highest level of education attained by the primary caregiver. Additionally, information on household marital status was included to capture family structure, with a particular focus on whether participants lived in single-parent or married households.

#### Environmental Exposure (PM2.5):

Exposure to fine particulate matter (PM2.5) was drawn from residential history data of the ABCD study. This variable is estimated using zip code and is available in the ABCD data linked to participants' residential addresses. The source of the PM2.5 data in Census and ABCD is the Environmental Protection Agency's Air Quality System, providing annual averages of PM2.5 concentrations. This variable was selected as a proxy of air pollution effect on neurodevelopment.

#### Resting-State Functional Connectivity (rsFC):

Neuroimaging data from the baseline ABCD study were utilized to examine resting-state functional connectivity (rsFC) between the right pallidum and the ventral attention network (rsfmri_cor_ngd_vta_scs_plrh). Functional Magnetic Resonance Imaging (fMRI) scans were analyzed to identify patterns of connectivity, focusing on regions implicated in cognitive control, reward processing, and attentional regulation.

#### Inhibitory Control:

Inhibitory control in this study was based on Positive Urgency. Positive urgency was measured using UPPS-SS [31]. Positive urgency is a construct that reflects an aspect of impulsivity. In this study, positive urgency (upps_y_ss_positive_urgency) was treated as a continuous measure, with a higher score indicating higher positive urgency traits (higher impulsivity) [32]. The UPPS-SS is a valid and reliable measure [32].

#### Substance Use Initiation:

Early substance use initiation was assessed based on self-reported data from the baseline ABCD study. Participants were asked about any history of tobacco use, including cigarettes and e-cigarettes, to identify individuals who had initiated substance use during early adolescence.

#### Race/Ethnicity.

Race/ethnicity was identified by the parents was a categorical variable: Black/African American, Asian, Latino, other/mixed race, and White (reference group).

### 2.4. Analytical Approach

The primary analytical method employed was Structural Equation Modeling (SEM), conducted using Stata software. SEM is a multivariate statistical technique that allows for the simultaneous examination of multiple relationships between variables, providing a comprehensive analysis of direct and indirect pathways. In this study, SEM was used to model the relationships between SES (using parental education and household structure), PM2.5 exposure, rsFC between the right pallidum and the ventral attention network P-(VAN rsFC), inhibitory control, and substance use initiation.

The SEM approach enabled the testing of complex mediational pathways, evaluating whether PM2.5 exposure and neurobiological factors mediated the relationship between SES and early substance use. Model fit indices, such as the Root Mean Square Error of Approximation (RMSEA), Comparative Fit Index (CFI), and Standardized Root Mean Square Residual (SRMR), were used to assess the adequacy of the SEM model. Missing data were handled using full information maximum likelihood estimation, allowing for the inclusion of all available data points. Sensitivity analyses were performed to ensure robustness, examining the consistency of findings across different subsamples, including demographic variables like gender and age.

The Institutional Review Board (IRB) is an ethics committee responsible for ensuring that research involving human participants is conducted ethically and safely. The IRB reviews study protocols to make sure that the rights, welfare, and privacy of participants are protected. For the ABCD study, the IRB approvals were obtained from participating institutions across the United States, ensuring that all aspects of data collection, participant interaction, and analysis were in compliance with ethical standards. This included informed consent procedures, protection of participant confidentiality, and adherence to guidelines for conducting research with minors. All participants and their legal guardians provided written informed consent and assent, ensuring that they were fully informed about the study's goals, procedures, potential risks, and benefits before participation.

## 3. Results

The results of the structural equation modeling (SEM) are summarized in Table 1, highlighting the associations between demographic, environmental, neurobiological, and behavioral factors related to PM2.5 exposure, rsFC between the right pallidum and the ventral attention network, inhibitory control, and substance use initiation.

Parental education (β = −0.100, SE = 0.011, 95% CI: −0.121, −0.079, p < 0.001) was significantly associated with lower PM2.5 exposure, indicating that higher levels of parental education corresponded to reduced exposure to PM2.5. Household structure also showed an influence, with youth from married households having lower PM2.5 exposure (β = −0.039, SE = 0.011, 95% CI: −0.059, −0.018, p < 0.001). In terms of racial and ethnic groups, Asian (β = 0.051, SE = 0.009, 95% CI: 0.033, 0.069, p < 0.001), Latino (β = 0.099, SE = 0.011, 95% CI: 0.078, 0.120, p < 0.001), and Black (β = 0.196, SE = 0.011, 95% CI: 0.175, 0.218, p < 0.001) individuals exhibited higher levels of PM2.5 exposure compared to other groups. Youth categorized as “Other” race also experienced significantly elevated PM2.5 exposure (β = 0.098, SE = 0.010, 95% CI: 0.079, 0.117, p < 0.001). Age (β = 0.000, SE = 0.009, 95% CI: −0.018, 0.018, p = 0.984) and gender (male) (β = −0.014, SE = 0.009, 95% CI: −0.032, 0.005, p = 0.140) were not significantly associated with PM2.5 exposure.

Higher levels of PM2.5 exposure were associated with reduced rsFC between the right pallidum and the ventral attention network (β = −0.025, SE = 0.010, 95% CI: −0.045, −0.006, p = 0.012). Age was positively related to increased rsFC between the right pallidum and the ventral attention network (β = 0.020, SE = 0.004, 95% CI: 0.012, 0.029, p < 0.001), indicating a potential age-related enhancement in connectivity. Male gender (β = −0.023, SE = 0.010, 95% CI: −0.042, −0.004, p = 0.018) was associated with lower rsFC. Parental education (β = −0.017, SE = 0.010, 95% CI: −0.037, 0.003, p = 0.097) and household marital status (β = 0.000, SE = 0.011, 95% CI: −0.020, 0.021, p = 0.978) did not show significant associations with rsFC.

Reduced rsFC between the right pallidum and the ventral attention network was significantly linked to lower inhibitory control (β = 0.112, SE = 0.011, 95% CI: 0.091, 0.134, p < 0.001), suggesting that altered connectivity is associated with poorer cognitive regulation.

Lower rsFC between the right pallidum and the ventral attention network was weakly associated with a higher likelihood of substance use initiation (β = −0.024, SE = 0.012, 95% CI: −0.048, 0.000, p = 0.050). Low inhibitory control was significantly associated with substance use initiation (β = −0.057, SE = 0.027, 95% CI: −0.111, −0.004, p = 0.037), indicating that youth with poorer inhibitory control were more likely to initiate substance use. Age was positively associated with substance use initiation (β = 0.049, SE = 0.004, 95% CI: 0.041, 0.056, p < 0.001), suggesting that older adolescents are more likely to begin using substances. Parental education was inversely related to substance use initiation (β = −0.088, SE = 0.011, 95% CI: −0.110, −0.066, p < 0.001), indicating that higher parental education was protective against early substance use. Male gender (β = −0.013, SE = 0.012, 95% CI: −0.035, 0.010, p = 0.265) was not significantly associated with substance use initiation.

**Figure 1.**
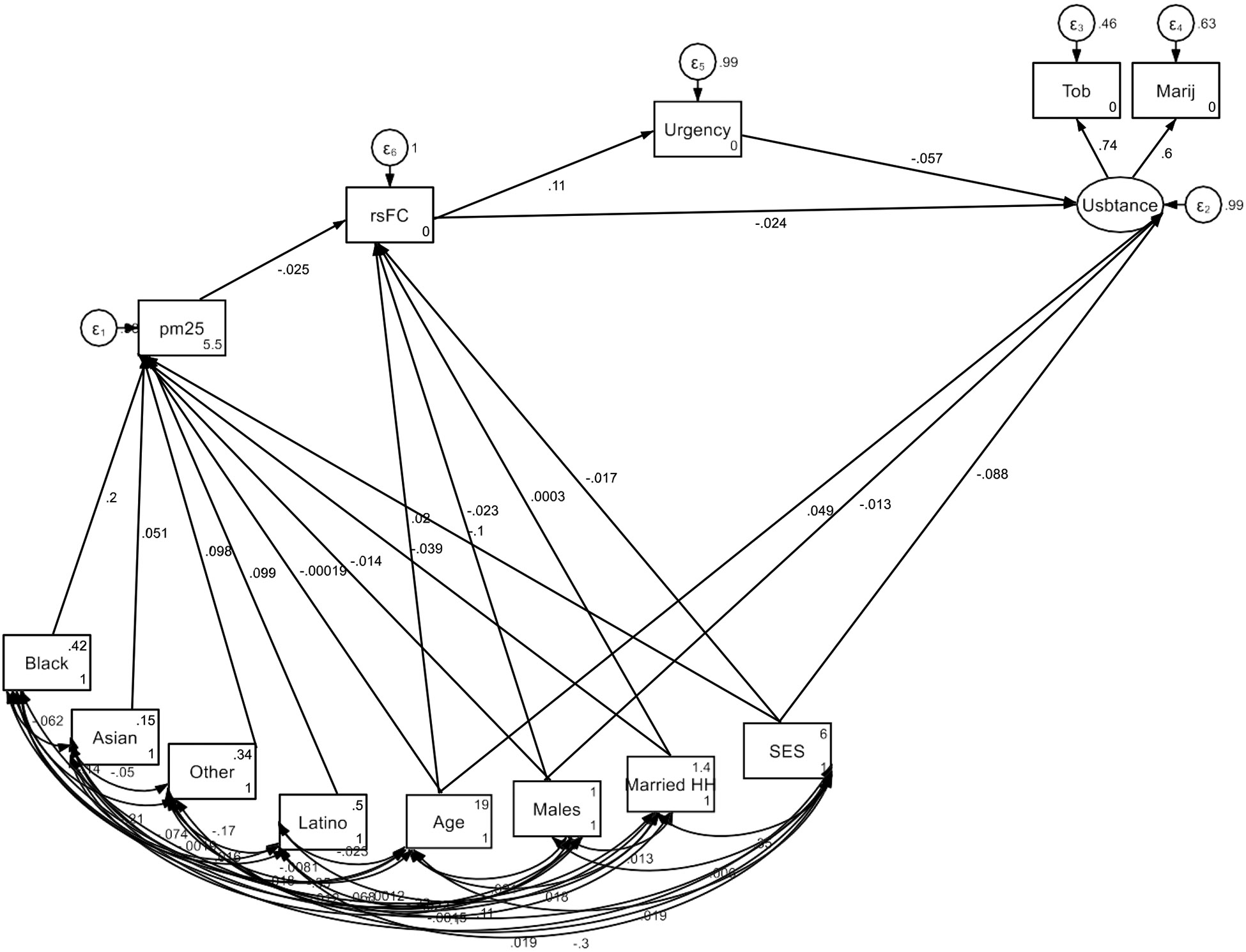
Summary of structural equation modeling (SEM)

Overall, the SEM results indicate a pathway where low SES, indicated by lower parental education and single-parent households, is linked to higher PM2.5 exposure. In turn, elevated PM2.5 exposure is associated with altered neurobiological functioning—specifically, reduced rsFC between the right pallidum and the ventral attention network—which impacts inhibitory control. Low inhibitory control subsequently contributes to an increased risk of substance use initiation in youth (Figure 1).

## 4. Discussion

This study aimed to explore the pathways through which SES and environmental factors influence early tobacco and marijuana use initiation among adolescents, focusing on the roles of PM2.5 exposure, P-VAN rsFC, and inhibitory control. The findings provide support for a multi-step pathway where lower SES, particularly parental education, is linked to higher levels of PM2.5 exposure, which in turn influences neurobiological functioning and cognitive control. These results highlight the complexity of factors contributing to substance use initiation, suggesting that both environmental exposures and cognitive vulnerabilities play significant roles.

### 4.1. Socioeconomic Status and PM2.5 Exposure

One of the most robust findings in this study is the relationship between SES indicators, such as parental education, and PM2.5 exposure. Lower levels of parental education were significantly associated with higher PM2.5 exposure, highlighting the persistent socioeconomic disparities in environmental risks. This association is likely driven by a combination of factors, including residential segregation, limited access to resources, and fewer opportunities for individuals in lower SES brackets to reside in areas with lower environmental pollutants. The finding underscores the need to address structural determinants of health that place individuals from lower SES backgrounds at a disproportionate risk of exposure to harmful environmental factors. This pathway emphasizes that interventions aimed at reducing SES disparities could indirectly lower PM2.5 exposure, potentially influencing a cascade of neurobiological and behavioral outcomes [1].

### 4.2. PM2.5 Exposure and rsFC Between the Right Pallidum and the Ventral Attention Network

The second key pathway explored in this study centers on the impact of PM2.5 exposure on brain function, specifically the rsFC between the right pallidum and the ventral attention network (P-VAN rsFC). Higher levels of PM2.5 exposure were found to be associated with altered connectivity in this network, highlighting how environmental pollutants may directly influence neurodevelopment. The pallidum, part of the basal ganglia, plays a crucial role in cognitive control, reward processing, and impulse regulation. Its connectivity with the ventral attention network, which supports attentional shifts and processing of salient information, is integral to behavioral regulation. The observed reduction in P-VAN rsFC due to increased PM2.5 exposure suggests that pollutants might impair the neurobiological systems responsible for inhibitory control. This pathway emphasizes the importance of addressing environmental risk factors, particularly air quality, to mitigate potential neurodevelopmental impacts that can contribute to risk-taking behaviors in adolescence.

### 4.3. rsFC and Inhibitory Control

A critical cognitive mechanism in the observed pathways is inhibitory control, a key factor in adolescent decision-making and impulse regulation. In this study, lower rsFC between the right pallidum and the ventral attention network (P-VAN rsFC) was associated with reduced inhibitory control. This relationship suggests that disruptions in functional connectivity may undermine cognitive processes necessary for controlling impulsive actions, making adolescents more susceptible to substance use. The association between disrupted P-VAN rsFC and reduced inhibitory control underscores the importance of understanding how environmental and neurobiological factors jointly shape cognitive vulnerabilities. Targeting inhibitory control through cognitive and behavioral interventions might serve as a potential strategy to reduce substance use risk, especially for those exposed to environmental pollutants.

### 4.4. Inhibitory Control and Substance Use Initiation

The final link in the pathway involves the impact of inhibitory control on substance use initiation. The study found that adolescents with lower inhibitory control were more likely to initiate tobacco and marijuana use, highlighting the well-established role of impulse regulation in substance-related behaviors. Reduced inhibitory control may lead to greater susceptibility to environmental and social cues that promote substance use, contributing to early initiation. This finding supports existing literature suggesting that cognitive control is a central mechanism in the development of risky behaviors during adolescence. Interventions that enhance inhibitory control could be particularly beneficial in preventing substance use among at-risk youth, reinforcing the need for programs that promote cognitive resilience.

### 4.5. Future Research Directions

Future research should further explore the mechanisms through which PM2.5 and other environmental pollutants affect neurodevelopmental processes and cognitive functioning. Longitudinal studies are needed to establish the temporal relationship between PM2.5 exposure, changes in P-VAN rsFC, and inhibitory control to better understand how these factors interact over time to influence substance use behaviors. Additionally, there is a need to examine potential protective factors, such as social support, community resources, and individual resilience, that may buffer the negative effects of low SES and environmental pollutants. Future studies could also investigate how interventions targeting cognitive control, such as training programs that enhance inhibitory skills, might mitigate the risk of early tobacco and marijuana use, particularly among those in high-risk environments.

### 4.6. Implications for Public Health and Policy

The findings of this study have significant implications for public health and policy. Addressing socioeconomic and environmental disparities should be a priority in efforts to prevent substance use among adolescents. Policies aimed at reducing environmental pollutants, particularly in low SES communities, could have far-reaching benefits for adolescent health and development. Improving air quality through stricter pollution regulations and urban planning initiatives could reduce the neurodevelopmental risks associated with PM2.5 exposure. Additionally, public health strategies should focus on early interventions that enhance cognitive resilience, particularly in children from socioeconomically disadvantaged backgrounds. Programs that promote parental education, cognitive skills development, and access to safe environments could play a crucial role in reducing substance use initiation.

### 4.7. Limitations

Despite the strengths of this study, several limitations must be acknowledged. First, the cross-sectional nature of the data limits the ability to make causal inferences about the observed relationships. Longitudinal studies are needed to clarify the directionality of the pathways and establish causality. Second, while PM2.5 exposure was used as a measure of environmental risk, it is only one of many environmental factors that may influence neurodevelopment and behavior. Future studies should incorporate additional environmental variables, such as noise pollution, neighborhood safety, and access to green spaces, to provide a more comprehensive picture of how environmental contexts shape adolescent risk behaviors. Third, the study focused on specific brain networks and regions; other neural circuits involved in cognitive control and reward processing were not examined, potentially limiting the scope of the findings. P-VAN rsFC is not the only connectivity that may have implications in linking low SES, low inhibitory control, and substance use. Future research should adopt a broader neurobiological perspective to capture the full range of brain mechanisms that contribute to adolescent substance use initiation.

This study contributes to a growing body of research highlighting the multifaceted nature of adolescent tobacco use initiation, demonstrating that it is influenced by an intricate interplay between socioeconomic, environmental, and neurobiological factors. Lower SES, through increased exposure to PM2.5, appears to disrupt brain connectivity patterns that are crucial for cognitive control, ultimately increasing the risk of early tobacco use. These findings suggest that effective prevention strategies need to be multi-dimensional, addressing both environmental exposures and cognitive vulnerabilities. Acknowledging the role of environmental pollutants and cognitive control mechanisms can help inform more comprehensive approaches to reducing substance use initiation in adolescents, especially those from socioeconomically disadvantaged backgrounds.

## 5. Conclusion

In conclusion, this study provides evidence for a multi-step pathway linking low SES to adolescent tobacco and marijuana use initiation, emphasizing the importance of both environmental and cognitive factors (P-VAN rsFC). Addressing a comprehensive approach—through environmental policy, targeted interventions, and support for cognitive development—may offer a more effective approach to preventing early substance use, particularly in vulnerable populations.

## Figures and Tables

**Table 1.** Summary of structural equation modeling (SEM)

Predictor		Outcome	B	SE	95%		CI
Age	→	PM2.5	0.000	0.009	−0.018	0.018	0.984
Male	→	PM2.5	−0.014	0.009	−0.032	0.005	0.140
Parental education	→	PM2.5	−0.100	0.011	−0.121	−0.079	< 0.001
Asian	→	PM2.5	0.051	0.009	0.033	0.069	< 0.001
Married household	→	PM2.5	−0.039	0.011	−0.059	−0.018	< 0.001
Other	→	PM2.5	0.098	0.010	0.079	0.117	< 0.001
Latino	→	PM2.5	0.099	0.011	0.078	0.120	< 0.001
Black	→	PM2.5	0.196	0.011	0.175	0.218	< 0.001
PM2.5	→	rsFC between the right pallidum and ventral attention network	−0.025	0.010	−0.045	−0.006	0.012
Age	→	rsFC between the right pallidum and ventral attention network	0.020	0.004	0.012	0.029	< 0.001
Male	→	rsFC between the right pallidum and ventral attention network	−0.023	0.010	−0.042	−0.004	0.018
Parental education	→	rsFC between the right pallidum and ventral attention network	−0.017	0.010	−0.037	0.003	0.097
Married household	→	rsFC between the right pallidum and ventral attention network	0.000	0.011	−0.020	0.021	0.978
rsFC between the right pallidum and ventral attention network	→	Low Inhibitory Control	0.112	0.011	0.091	0.134	< 0.001
rsFC between the right pallidum and ventral attention network	→	Substance Use Initiation	−0.024	0.012	−0.048	0.000	0.050
Low Inhibitory Control	→	Substance Use Initiation	−0.057	0.027	−0.111	−0.004	0.037
Age	→	Substance Use Initiation	0.049	0.004	0.041	0.056	< 0.001
Male	→	Substance Use Initiation	−0.013	0.012	−0.035	0.010	0.265
Parental education	→	Substance Use Initiation	−0.088	0.011	−0.110	−0.066	< 0.001
